# The value of endocervical curettage during large loop excision of the transformation zone in combination with endocervical surgical margin in predicting persistent/recurrent dysplasia of the uterine cervix: a retrospective study

**DOI:** 10.1186/s12905-024-03291-w

**Published:** 2024-08-21

**Authors:** Anne Cathrine Scherer-Quenzer, Jelena Findeis, Saskia-Laureen Herbert, Nithya Yokendren, Ann-Kristin Reinhold, Tanja Schlaiss, Achim Wöckel, Joachim Diessner, Matthias Kiesel

**Affiliations:** 1 Department of Obstetrics and Gynecology, University Hospital of Wuerzburg, Josef-Schneider-Strasse 4, 97080 Wuerzburg, Germany; 2 Cardiology Division, Kantonsspital St Gallen, Rorschacher Strasse 95, St. Gallen, 9007 Switzerland; 3 Department of Anesthesiology, University Hospital of Wuerzburg, Oberduerrbacher Strasse 6, 97080 Wuerzburg, Germany

**Keywords:** ECC, Residual disease, Risk of recurrence, Risk factors, Cervical cancer, LLETZ

## Abstract

**Background:**

Cervical cancer often originates from cervical cell dysplasia. Previous studies mainly focused on surgical margins and high-risk human papillomavirus persistence as factors predicting recurrence. New research highlights the significance of positive findings from endocervical curettage (ECC) during excision treatment. However, the combined influence of surgical margin and ECC status on dysplasia recurrence risk has not been investigated.

**Methods:**

In this retrospective study, data from 404 women with high-grade squamous intraepithelial lesions (HSIL) who underwent large loop excision of the transformation zone (LLETZ) were analyzed. Records were obtained retrospectively from the hospital’s patient database including information about histopathological finding from ECC, endocervical margin status with orientation of residual disease after LLETZ, recurrent/persistent dysplasia after surgical treatment and need for repeated surgery (LLETZ or hysterectomy).

**Results:**

Patients with cranial (= endocervical) R1-resection together with cells of HSIL in the ECC experienced re-surgery 17 times. With statistical normal distribution, this would have been expected to happen 5 times (*p* < 0.001). The Fisher’s exact test confirmed a statistically significant connection between the resection status together with the result of the ECC and the reoccurrence of dysplasia after surgery (*p* < 0,001). 40,6% of the patients with re-dysplasia after primary LLETZ had shown cranial R1-resection together with cells of HSIL in the ECC. Investigating the risk for a future abnormal Pap smear, patients with cranial R1-resection together with dysplastic cells in the ECC showed the greatest deviation of statistical normal distribution with SR = 2.6.

**Conclusion:**

Our results demonstrate that the future risk of re-dysplasia, re-surgery, and abnormal Pap smear for patients after LLETZ due to HSIL is highest within patients who were diagnosed with cranial (endocervical) R1-resection and with cells of HSIL in the ECC in their primary LLETZ. Consequently, the identification of patients, who could benefit of intensified observation or required intervention could be improved.

## Background

Cervical cancer is the fourth most common cancer among women worldwide, with 604,127 new cases reported in 2020 [1]. This malignancy typically arises from cervical cell dysplasia, known as cervical intraepithelial neoplasia (CIN) [2]. The incidence of CIN is approximately 30–100 times higher than that of cervical cancer [3].

Early detection programs can significantly improve prognosis by identifying precancerous lesions and early-stage tumors [4]. In Germany, these programs include Pap smears and human papillomavirus (HPV) testing. HPV, a common sexually transmitted virus, is responsible for over 95% of all cervical cancer cases (WHO fact sheet on cervical cancer). If cervical dysplasia is suspected, colposcopy, possibly accompanied by biopsy or endocervical curettage (ECC) as recommended by the Society for Colposcopy and Cervical Pathology, is performed. If high-grade squamous intraepithelial lesion (HSIL) is diagnosed, surgical intervention, such as large loop excision of the transformation zone (LLETZ), is indicated. Despite the surgical technique used, there remains a risk of recurrent or persistent dysplasia, with residual or recurrent HSIL occurring in 5–25% of patients [5].

Therefore, participation in appropriate aftercare programs is particularly important [6]. Recurrent cervical intraepithelial dysplasia is most frequently diagnosed within the first two years post-surgery, which is why diagnostic examinations in Germany is recommended at 6-, 12-, and 24-months post-intervention. Following the early detection program, the aftercare protocol includes a combination of Pap smear and HPV testing (German Cancer Guidelines 2020). For patients with R1 resection margins, the risk of recurrence is up to four times higher than for those with clear margins [7]. If both the Pap smear and HPV test remain negative after two years, normal screening intervals can be resumed. However, if either test is positive, a colposcopy should be performed.

Recent studies suggest that administering a HPV vaccine, either shortly before or after surgical treatment for CIN II or CIN III, may reduce the risk of recurrence [8]. Regarding the development of lower genital tract dysplasia, the estimated potential protective effect of vaccination was 67%, supporting HPV vaccination in patients receiving treatment for HPV-related diseases [9]. Nevertheless, another meta-analysis indicated that vaccinating 45.5 women, either before or after conization, is necessary to prevent one case of recurrent CIN II or CIN III [10]. Therefore, further research is needed to evaluate the effectiveness of implementing vaccination in this context.

Up til now, positive surgical cervical margins and persistence of high-risk HPV are the main known factors predicting the risk of recurrence [11]. New studies indicate that positive findings from ECC, performed during excision treatment, had a higher influence on the predictions of persistent/recurrent intracervical dysplasia than the status of the surgical cervical margins [12]. To our knowledge, there is no study that investigated the combined influence of surgical margin and status of endocervical curettage on the risk of recurrent/persistent intracervical dysplasia.

## Methods

In this retrospective study, data from 404 women with high-grade squamous intraepithelial lesions (HSIL) who underwent large loop excision of the transformation zone (LLETZ) were analyzed. The purpose of this study is to help optimize the diagnostic procedure during and after LLETZ due to HSIL.

### Patient selection

All patients presenting for an examination to the special dysplasia consultation hour of the Department of Obstetrics and Gynecology at the University Hospital Wuerzburg between 1st of January, 2016 and 31st of March, 2021 were considered. These patients were listed with their patient ID (identification number) in Excel spreadsheets and could thus be accessed via the hospital’s internal information system. The digital medical records were reviewed using the hospital’s software. The required data including information about histopathological findings from ECC, endocervical margin status with orientation of residual disease after LLETZ, recurrent/persistent dysplasia after surgical treatment and need for repeated surgery (LLETZ or hysterectomy) were extracted retrospectively from medical history forms, doctor’s letters, surgical reports, as well as pathology, virology, and microbiology findings. In ten cases, attempts were made by telephone to obtain findings from previous conizations. The data collection was completed with the end date of March 2021. Due to this, later findings of the patients could not be considered. All patients were anonymized for this study using their patient ID.

### Patient characteristics

Patients were included based on the following criteria: LLETZ with high-grade squamous intraepithelial lesion performed at the Department of Obstetrics and Gynecology (University of Wuerzburg) for which follow-up information was available. The primary LLETZ and re-surgery was carried out by three specialists for gynecologic cancer with at least 10 years of experience in conization.

Patients were excluded from this study based on the following exclusion criteria:

- No LLETZ was performed (28 patients),
- No CIN was detected or the histopathological finding was not usable (52 patients),
- Only low-grade and no high-grade squamous intraepithelial lesion was detected after LLETZ (6 patients),
- Cervical cancer was detected (41 patients, with 10 adenocarcinomas, 28 squamous cell carcinomas and three less common forms of carcinoma: two microinvasive squamous cervical cancer and one neuroendocrine carcinoma of the cervix),
- No data was available on a previously performed LLETZ (8 patients).

If HSIL as well as LSIL were detected simultaneously, only the HSIL and its resection status was considered. In total, we reviewed 404 cases of LLETZ with HSIL, 135 patients had to be excluded for the reasons mentioned above.

### Human patient data

This retrospective study was registered with the Institutional Review Board (Ref. No. 20200911-01).

### Important definitions/explanations

At the Department of Obstetrics and Gynecology at the University Hospital Wuerzburg LLETZ is typically performed in conjunction with endocervical curettage (ECC). This is in line with the recommendations of the German Society for Colposcopy and Cervical Pathology [13]. The cytological appearance of moderate dysplasia, analogous to CIN II, and severe dysplasia, analogous to CIN III, was classified as HSIL.The status of endocervical margins was defined as positive in case of microscopic involvement of dysplasia (HSIL) into to resection margins. The study focuses on the following resection margins: R0, R1 cran., R1 cran. with ECC and “others”: R0 means that all of the high-grade squamous intraepithelial lesion have been completely removed, with no dysplastic cells detected in the pathological results of the endocervical curettage. R1 cran. is defined as the presence of HSIL cells within the cranial (endocervical) resection margin, indicating that HSIL was found at the edge of the excised tissue. R1 cran. with ECC means that HSIL cells are found both within the cranial (endocervical) resection margin and in the ECC results, indicating residual HSIL in both the resection margin and the endocervical sample. “Others” encompasses cases where the resection status is classified as R1 lateral or Rx, which includes situations where HSIL is present in the lateral margins or where the resection status is uncertain.

The status of endocervical curettage was classified as positive, if it contained dysplastic squamous epithelium. Patients were considered to have persistent/recurrent disease, if histological examination after repeated surgery showed any evidence of HSIL.

### Statistical analysis

Statistical analysis was performed using SPSS version 26 and 28 for Windows (SPSS Inc., Chicago, IL. USA). All *p* values ≤ 0.05 were considered statistically significant. Normally distributed variables were expressed as mean ± SD, categorical variables were expressed as number (percentage). T- test and Mann-Whitney test were used for analysis of quantitative data and comparison as appropriate. Analysis of categorical data was undertaken using Chi-square test or Fisher’s exact test. The effect sizes of Chi-square test and Fisher’s exact test were individually calculated using Cramer’s V with values ≥ 3 or greater being considered moderately strong effects and values ≥ 5 or greater being considered strong effects. Logistic regression was used to estimate the relationship of findings from ECC and endocervical margin status with the occurrence of recurrent/persistent dysplasia or need for repeated surgery. The odds ratio (an estimate of the relative risk of persistent/recurrent disease) and the 95% confidence interval were calculated for each variable.

### Use of large language models

ChatGPT Version 4.0 (OpenAI Inc., San Francisco) was used for language quality check.

## Results

### Resection status and ECC

All of the 404 patients showed cells of a HSIL in the pathologic results of the LLETZ. 36 of the patients also showed cells of a HSIL in the ECC. The pathologic results of the ECC could not be evaluated in the case of nine patients. With four patients, no ECC had been performed during surgery. 264 patients (65.3%) showed R0 resection. 39 patients (9.7%) showed HSIL in the cranial (= endocervical) resection margin (= R1 cran.). Another 34 patients (8.4%) had pathologic results showing HSIL in the cranial resection margin as well as in the ECC (= R1 cran. with ECC). 67 patients (16.6%) had one of the following resection status: R1 lateral and Rx (uncertain resection status). These were summarized as “Others” and underwent no further statistical examination. The frequency of the different resection status after surgery is displayed in Table 1.

**Table 1:** Amount of different resection status

Resection status	Amount	Percent (%)
**R0**	264	65.3
**R1 cran.**	39	9.7
**R1 cran. with ECC**	34	8.4
**Others**	67	16.6
**Sum**	404	100.0

R0: all of the HSIL has been removed with no cells of a HSIL in the ECC. R1 cran.: Cells of a HSIL are found within the cranial (= endocervical) resection margin. R1 cran. with ECC: Cells of a HSIL are found within in the cranial (= endocervical) resection margin as well as in the ECC. “Others” incorporates the following resection status: R1 lateral and Rx (uncertain resection status)

### The frequency of secondary surgery after LLETZ

Of 404 patients, 58 (14,4%) experienced re-surgery. This was done via LLETZ in 28 and via hysterectomy in 30 cases. In the case of 2 patients, a third surgery was performed. Patients with cranial (= endocervical) R1-resection together with cells of HSIL in the ECC experienced re-surgery 17 times. With statistical normal distribution, this would have been expected to happen 5 times, showing a statistically significant difference (*p* < 0.001). Patients with cranial (= endocervical) R1-resection without cells of HSIL in the ECC had a re-LLETZ or hysterectomy in 13 cases, versus 6 expected re-surgeries in case of normal distribution. This difference was also significant (*p* < 0.001). 13 patients with R0-resection and no cells of HSIL in the ECC, experienced re-surgery versus the amount of expected 38 patients with normal distribution (*p* < 0.001). The Chi-Square Test was used to show that all the above-described differences were statistically significant. Cramer’s V showed a moderately strong result (V = 0.41). Table 2 contains an overview of the described resection status after primary LLETZ as well as the histological results of the ECC together with the according amounts of re-surgeries.

**Table 2:** Amount of patients with re-surgery according to the resection status of the primary LLETZ and ECC

Resection status primary surgery			Re-surgery		Sum
			Yes	No	
	**R0**	Amount	13*	251	264
		Expected	38*	226	264
		% of R0	4.9%	95.1%	100.0%
		SR	-4.0	1,7	
	**R1 cran.**	Amount	13*	26	39
		Expected	6*	33	39
		% of R1 cran.	33.3%	66.7%	100.0%
		SR	3.1	-1.3	
	**R1 cran. with ECC**	Amount	17*	17	34
		Expected	5*	29	34
		% of R1 cran. with ECC	50.0%	50.0%	100.0%
		SR	5.5	-2.2	
	**Others**	Amount	15	52	67
		Expected	10	57	67
		% of others	21.4%	77.6%	100.0%
		SR	1.7	-0.7	
**Sum**		Amount	58	346	404
		% of Sum	14.4%	85.6%	100.0%

As described in the section “Methods”, Standardized Residuals (SR) describe the difference between the observed amount and the amount in case of statistical independence between e.g. resection status and re-surgeries. The larger the difference, the larger the probability of a statistically significant connection between these two variables. Re-surgery = Re-LLETZ or hysterectomy, with ECC = cells of HSIL found in the tissue of the ECC during performance of LLETZ, Expected = expected amount in case of statistical independence, *=statistically significant difference in respective group (*p* < 0.001). The Chi-Square Test was used for testing statistically significant differences

### The rate of recurrent dysplasia in re-surgery depending on ECC status and resection margin

36 patients showed cells of HSIL in the ECC during LLETZ-procedure, independently of the resection status. 13 of these patients (36.1%) developed re-dysplasia in the wake of time. 355 patients showed no cells of HSIL in the ECC. Of this population, only 9 patients (5.1%) experienced re-dysplasia. With a SR of 6.0, it was more likely to find re-dysplasia in case of cells of HSIL in the ECC during LLETZ-procedure. With a SR of -2.0 it was more unlikely to detect re-dyplasia, if the ECC had shown no HSIL, compared to statistical normal distribution. Fisher’s exact test depicted a statistically significant connection between the histological result of the ECC during LLETZ and the occurrence of re-dysplasia (*p* < 0.001). Cramer’s V showed a moderately strong result for this finding (V = 0.33).

32 patients were diagnosed with re-dysplasia or persistent dysplasia after primary LLETZ. 13 (38.2% of these patients had shown cranial (= endocervical) R1-resection together with cells of HSIL in the ECC. 6 patients (15.4%) had shown cranial R1-resection without cells of HSIL in the ECC. 9 patients (3.4%) had R0-resection and 4 patients (6.0%) had a resection status classified as “other” (= R1 lateral, R1 lateral and cranial or Rx). Patients with R1-resection suffered from re-dysplasia more often than it would to be expected in case of statistical normal distribution (SR = 6.3). Additionally, patients with R0-resection showed re-dysplasia less often than in a population with normal distribution (SR = -2.6). The Fisher’s exact test confirmed a statistically significant connection between the resection status together with the result of the ECC and the reoccurrence of dysplasia after surgery (*p* < 0.001). Cramer’s V displayed a moderately strong result (V = 0.36). This is further depicted in Table 3.

**Table 3:** Amount of patients with re-dysplasia after LLETZ, sorted by resection status and ECC

			Re-dysplasia		Sum
			Yes	No	
**Resection status**	**R0**	Amount	9*	255	264
		Expected	21*	243	264
		% of R0	3.4%	96.6%	100.0%
		SR	-2.6	0.8	
	**R1 cran.**	Amount	6*	33	39
		Expected	3*	36	39
		% von R1 cran.	15.4%	84.6%	100.0%
		SR	3.1	-1.3	
	**R1 cran. with ECC**	Amount	13*	21	34
		Expected	3*	31	34
		% of R1 cran. with ECC	38.2%	61.8%	100.0%
		SR	6.3	-1.8	
	**Others**	Amount	4	63	67
		Expected	5	62	67
		% of Others	6.0%	94.0%	100.0%
		SR	-0.6	0.2	
**Sum**		Amount	32	372	404
		% of Sum	7.9%	92.1%	100.0%

Re-dyslasia = cells of HSIL in re-surgery or ECC or biopsy after LLETZ, Expected = expected amount in case of statistical independence, SR = standardized residual, *=statistically significant difference in respective group (*p* < 0.001)

In addition, the Odds Ratio (OR) for the different resection status was evaluated, in order to further examine the risk of re-dysplasia. The reference category was R0 resection. The risk of re-dysplasia for every resection-status was compared to the risk of dysplasia with R0-resection. Cranial (= endocervical) R1-resection with no cells of HSIL in the ECC showed an Odds Ratio of 5.15 compared to R0-resection (95% CI, 1.72–15.40, *p* < 0.003). Cranial (= endocervical) R1-resection with cells of HSIL in the ECC lead to an OR of 17.54 (95% CI, 6.72–45.78, *p* < 0.001). Resection status classified as “other” showed no statistical significance concerning the OR (*p* < 0.341).

### The rate of recurrent dysplasia in Pap smear after LLETZ

In the following, a Pap smear result of Pap IIg (“AGC-NOS” according to Bethesda) and higher was determined as abnormal finding. 39 of 404 patients showed an abnormal Pap smear after LLETZ. Among these 39 patients, 8 patients (20.5%) showed cranial (= endocervical) R1-resection together with cells of HSIL in the ECC. 5 patients (12.8%) had cranial R1-resection with no cells of HSIL in the ECC. 19 patients (7.2%) experienced R0-resection and 7 patients (10.4%) presented resection margins classified as “other” (= R1 lateral, R1 lateral and cranial or Rx). Patients with cranial R1-resection together with dysplastic cells in the ECC showed the greatest deviation of statistical normal distribution with SR = 2.6, followed by patients with merely cranial R1-resection (SR = 0.6) and those with R0-resection (SR = -1.3). Fisher’s exact test confirmed these differences as statically significant (*p* < 0.024) and Cramer’s V showed a low result of 0.16. These findings are depicted in Table 4.

**Table 4:** Amount of patients with abnormal findings in pap smear results, sorted by resection status and ECC

			Pap smear		Sum
			Yes	No	
**Resection status**	R0	Amount	19*	245	264
		Expected	26*	238	264
		% von R0	7.2%	92.8%	100.0%
		SR	-1.3	0.4	
	R1 cran.	Amount	5*	34	39
		Expected	4*	35	39
		% of R1 cran.	12.8%	87.2%	100.0%
		SR	0.6	-0.2	
	R1 cran. with ECC	Amount	8*	26	34
		Expected	3*	31	34
		% of R1 cran. with ECC	23.5%	76.5%	100.0%
		SR	2.6	-0.9	
	Others	Amount	7	60	67
		Expected	6	61	67
		% of others	10.4%	89.6%	100.0%
		SR	0.2	-0.1	
**Sum**		Amount	39	365	404
		% of Sum	9.7%	90.3%	100.0%

Abnormal findings in Pap smear = from Pap IIg on. Expected = expected amount in case of statistical independence, SR = standardized residual, *=statistically significant difference in respective group (*p* < 0.05)

### Age

At the time of primary LLETZ the mean age was 36,85 years with a standard deviation of 9.08 years. Chi-square tests were conducted to determine whether there were differences in the risk of re-surgery or recurrent dysplasia among different age groups. Significant differences were only found with respect to the risk of re-surgery. It was observed that patients over 40 years of age experienced re-surgeries more frequently than expected, while patients under 35 years of age underwent re-surgeries less frequently than expected (Chi-square = 13.12; *n* = 5; *p* < 0.022 with Cramer’s V = 0.18; *p* < 0.022).

## Discussion

This work contributes to the improvement of aftercare for patients, who have experienced a LLETZ due to HSIL. It is well-known that persistent HPV infection in patients with high-grade squamous intraepithelial lesions undergoing cervical excision is strongly linked to the recurrence and progression of cervical dysplasia. Therefore, recent research has mainly focused on risk factors contributing to the persistence of HPV infection in patients with HSIL [14]. Yet, certain clinicopathological and physiological factors, including human papillomavirus 16, high viral load, age over 50 years and positive surgical margins, have prognostic significance for persistent HPV infection, consequently influencing patient outcome [15]. Until now, the role of an ECC during LLETZ and its pathological results have played a minor role in the aftercare.

### Frequency of re-dysplasia, re-LLETZ, re-surgery and abnormal Pap

The frequency of re-dysplasia after LLETZ within a 5-year follow-up is stated with 7.9% (32 of 404 patients) in this study. This is congruent to existing data [16–18]. The rate of re-LLETZ or hysterectomy can be found to vary from 2.5 to 12.0% in the literature [19–22]. In the presented work, the rate of re-surgery is 14.4%, indicating representative data. Same can be said about the frequency of abnormal Pap smear after LLETZ. 9.7% of the patients in this work were found to experience cytologically suspicious findings during aftercare, which is within the range of 6–18% found in other studies [6, 23–25]. Consequently, the presented data analysis appears to be comparable with prior studies, adequately representing the concerning patient population.

### The resection status

R1-resection together with abnormal Pap smear themselves are risk factors for dysplastic cells in the ECC after LLETZ. Cells of HSIL found in the ECC during aftercare are adequate risk factors for remaining or recurrent dysplasia after LLETZ, even if Pap smear or biopsy were negative [16, 18, 26, 27]. In congruence to this, especially cranial (= endocervical) R1-resection significantly increases the risk of re-dysplasia [5, 12, 18, 19]. In this work, a statistically significant connection between resection status and result of ECC and risk of re-dysplasia (*p* < 0.001), re-surgery (*p* < 0.001) and abnormal Pap smear results (*p* < 0.024) in the aftercare could be shown. The highest risk of re-surgery, re-dysplasia and abnormal Pap smear was found for those patients, who had experienced cranial (= endocervical) R1-resection together with cells of HSIL in the ECC. If the ECC was negative, this risk was lower, even if there was R1-resection. The patient group with complete R0-resection had the lowest risk for re-dysplasia, re-surgery and abnormal Pap smear.

The percentage of R1-resection after LLETZ in this work is 24.5% of cases. This is congruent to the 11% and 31% stated in the literature [17, 19, 24, 28–32]. In a work of Arbyn et al., patients with R1-resection had a relative risk of 4.8 for re-dysplasia compared to patients with R0-resection [17]. 9–22% of patients with R1-resection suffer from re-dysplasia [17, 33, 34]. In case of R0-resection, this was only 1–4% [17, 35, 36].

### The ECC

It has been proven that an ECC prior to LLETZ can provide information concerning endocervical dysplasia. An ECC showing cells of HSIL before surgery is associated with more R1-resections [37–40]. 3–13% of patients in the literature and 9,0% in this work show cells of HSIL in the ECC, if it is performed directly after LLETZ-procedure [12, 41, 42]. In this study, a strong correlation between pathological result of the ECC and risk of re-dysplasia was found. This is comparable to the existing data [12, 42, 43]. These results indicate that cells of HSIL in ECC are a superior prognostic factor to the cranial resection status concerning re-dysplasia. By combining both aspects, the diagnostic validity can be increased. This can be seen by comparing the OR of this study: The OR for re-dysplasia was 17.54 among patients with cranial (= endocervical) R1-resection and HSIL in the ECC. If the resection status was cranial R1, but the ECC was negative, the OR was only 5.15, compared to R0-resection with negative ECC. This supports the performance of ECC directly after every LLETZ-procedure, in order to evaluate the risk of re-dysplasia during aftercare.

### Strengths and limitations

While previous research has primarily concentrated on surgical margins and HPV persistence, this study explores how ECC results impact recurrence risk, adding a new dimension to understanding post-surgical outcomes. Although the sample size is substantial, it could have been larger. Although the sample size is substantial, it could have been larger. Patients with cervical cancer were excluded, limiting the number of eligible participants. Follow-up examinations are often conducted by external practitioners, resulting in incomplete follow-up data for some cases and their subsequent exclusion from the study. Additionally, the study included only patients who underwent surgery by an expert. These factors contributed to a lower observed case number. The retrospective nature of the study and the fact that it was conducted at a single institution may introduce selection bias. Therefore, the results may not be applicable to all populations, especially those outside the specific demographic and clinical context of the study cohort. Thus, the findings should be validated through further research in a multi-center setting to ensure broader applicability. Moreover, the study does not address HPV persistence and endocone depth, which are a well-established risk factor for recurrence. Incorporating those aspects would provide a more comprehensive risk assessment.

## Conclusion

Patients who have undergone LLETZ with cranial (endocervical) R1-resection and positive HSIL cells in the ECC are at a significantly higher risk of recurrent dysplasia. These patients should be closely monitored post-surgery with more thorough surveillance protocols. Yet, patients with negative ECC can be spared potentially unnecessary interventions and resources could be allocated differently. Educating patients about the increased risk of recurrence associated with positive ECC findings and incomplete resection can help in shared decision-making regarding their treatment and follow-up care. Interestingly, the German Society for Colposcopy and Cervical Pathology states, that the accuracy of colposcopy in general is enhanced by performing an endocervical curettage (ECC), without making a distinction between squamous cell carcinoma (SCC) or adenocarcinoma in situ (AIS) [13]. The identification of high-risk patients based on ECC results and resection status suggests that more individualized treatment plans are necessary. Consequently, research on additional treatment as well as preventive measures for these high-risk patients, in order to reduce the likelihood of recurrence, is needed. Moreover, future studies should compare the benefits of ECC in predicting redysplasia and the need for resurgery with the often routinely resected endocone. This comparison will help determine the most effective methods for assessing recurrence risk and guiding patient care.

## Data Availability

No datasets were generated or analysed during the current study.

## Abbreviations

ECC: Endocervical curettage
LLETZ: Large loop excision of the transformation zone
HPV: Human papilloma virus
